# Supplementary material for: ASH2L Aggravates Fibrosis and Inflammation through HIPK2 in High Glucose-Induced Glomerular Mesangial Cells
Source: Genes (Basel). 2022 Nov 29;13(12):2244. doi: 10.3390/genes13122244 (PMC9816940; doi:10.3390/genes13122244)
Supplement: Supplementary file 1 [file genes-13-02244-s001.zip › genes-2009244-supplementary-1.pdf]

genes

MDPI

Supplementary figure

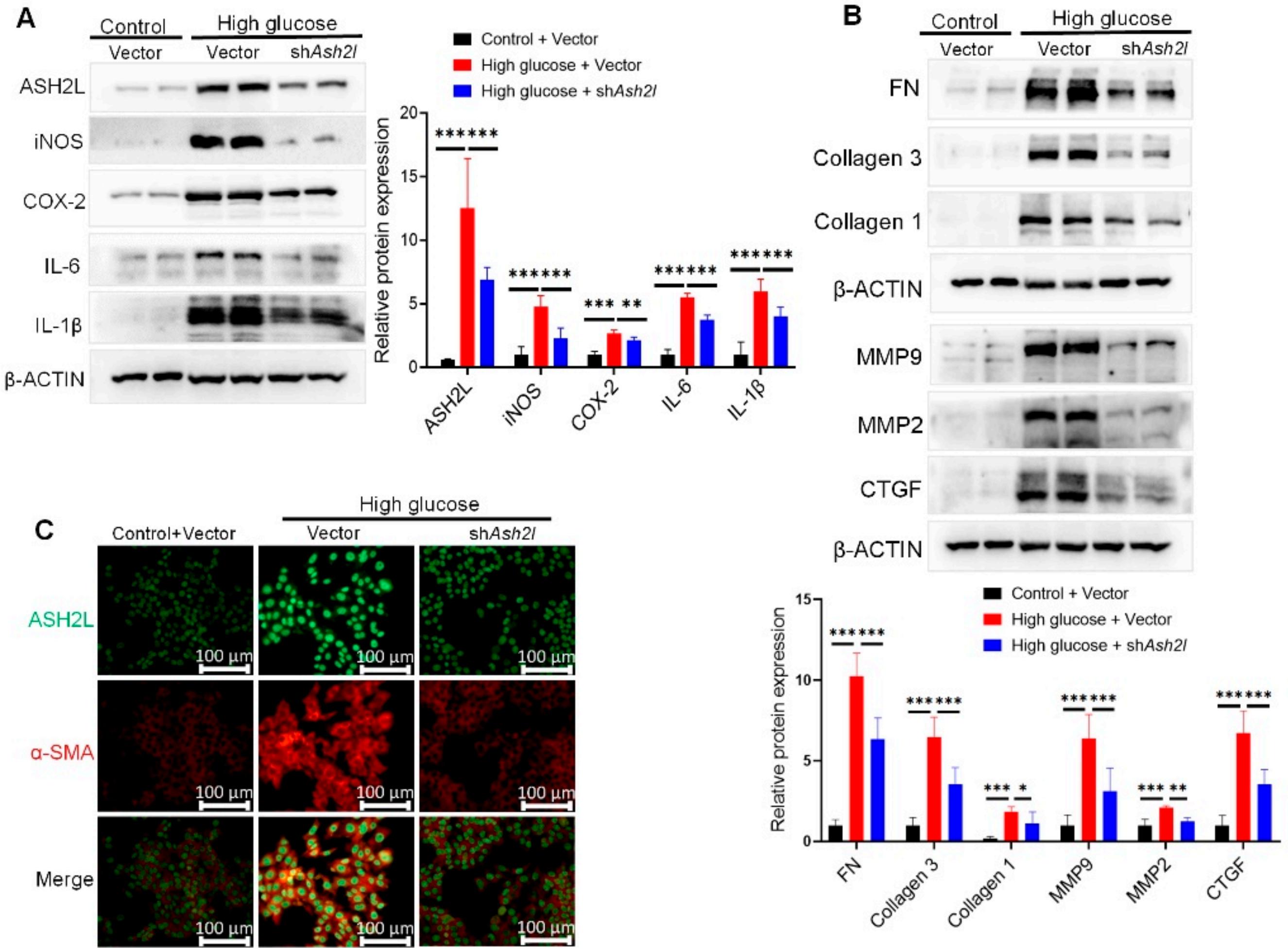

Figure S1. Knockdown of ASH2L decreases fibrosis and inflammation markers in mesangial cells. (**A**,**B**) Western blot analysis of ASH2L, inflammatory mediators (iNOS, COX-2, IL-6, IL-1β), and fibrosis markers (FN, Collagen 3, Collagen 1, MMP9, MMP2, CTGF) expression in SV40-MES-13 cells incubated with vector or ASH2L lentivirus (sh*Ash2l*) for 24 h, followed by 24 h of 33 mM high glucose treatment. Data from at least three independent experiments are shown as mean ± S.D, * $p < 0.05$, ** $p < 0.01$, and *** $p < 0.001$. (**C**) Immunofluorescence analysis of ASH2L and α-SMA in SV40-MES-13 cells calculated with vector or sh*Ash2l* for 24 h, followed by 24 h of 33 mM high glucose treatment.

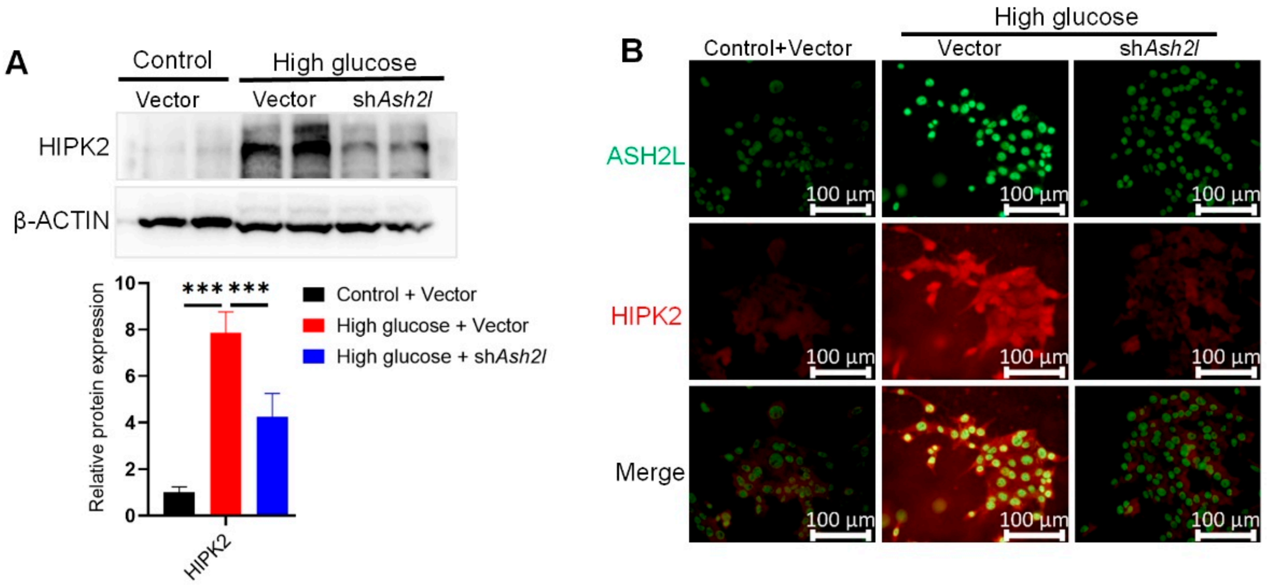

Figure S2. Loss of ASH2L suppresses HIPK2 expression in high glucose induced mesangial cells. (**A**) Western blot analysis of HIPK2 expression in SV40-MES-13 cells incubated with vector or ASH2L lentivirus (sh*Ash2l*) for 24 h, followed by 24 h of 33 mM high glucose treatment. Data is shown as mean ± S.D from at least three independent experiments, *** $p < 0.001$. (**B**) Immunofluorescence staining of ASH2L and HIPK2 in SV40-MES-13 cells.

Supplementary tables

Table S1. siRNA used for transfection.

siRNA	Sequence (5′ to 3′)
si*Ash2l*	GCUGCACGGUUUCCAUUAATT
	UUAAUGGAAACCGUGCAGCTT
si*Hipk2*	CACCCAUGAUUCAGAAUAATT
	CACCCAUGAUUCAGAAUAATT
siNC	UUCUCCGAACGUGUCACGUTT
	ACGUGACACGUUCGGAGAATT

Table S2. Primary antibodies used for immunoblots.

Primary antibodies	Company (Cat. No)	Source
FN	Servicebio (GB114491)	Rabbit
MMP9	Servicebio (GB12132)	Mouse
MMP2	Servicebio (GB11130)	Rabbit
α-SMA	Servicebio (GB111364)	Mouse
CTGF	Proteintech (25474-1-AP)	Rabbit
Collagen 1	Proteintech (14695-1-AP)	Rabbit
Collagen 3	Proteintech (22734-1-AP)	Rabbit
COX-2	Proteintech (27308-1-AP)	Rabbit
IL-6	Proteintech (21865-1-AP)	Rabbit
IL-1β	Proteintech (16806-1-AP)	Rabbit
iNOS	Cell Signal Technology (39898S)	Rabbit
ASH2L	Cell Signal Technology (5019T)	Rabbit
H3Kme3	Abcam (ab8580)	Rabbit
HIPK-2	Abcam (ab108543)	Rabbit
β-ACTIN	Proteintech (66009-1-Ig)	Mouse
H3	Cell Signal Technology (4499S)	Rabbit

Table S3. Primers used for qRT-PCR validation.

Gene name	Primer name	Primer sequence (5′ to 3′)
Ash2l	Mouse _ ASH2L _F	AGAAGGGAGGTCAACTGGAG
	Mouse _ ASH2L _R	CGCCTGGGTATCCATCACTT
Hipk2	Mouse _ HIPK-2 _F	ACACAGGCTCAAGATGGCAG
	Mouse _ HIPK-2 _R	ATGGCAACGGAGAAGGGAAC
Kmt2b	Mouse _ KMT2B _F	CAGTCCGTGGAGTTCTGGAC
	Mouse _ KMT2B _R	GCCTCGCCTGCTGAATAGTG
Kmt2d	Mouse _ KMT2D _F	ATCAAACAGGGTCGGAGCAG
	Mouse _ KMT2D _R	TCAGCCACCAGTGTCTCAAC
Cxxc1	Mouse _ CXXC1 _F	GATGCAGAAGAGTCCCCGTT
	Mouse _ CXXC1 _R	TCCTCCTTCTTCTTCTCGGACT
Col3a2	Mouse _ Collagen 3 _F	ACGTAAGCACTGGTGGACAG
	Mouse _ Collagen 3 _R	CAGGAGGGCCATAGCTGAAC
Mmp9	Mouse _ MMP9 _F	CAGACGTGGGTCGATTCCAA
	Mouse _ MMP9 _R	TCATCGATCATGTCTCGCGG
Mmp2	Mouse _ MMP2 _F	AGCTGTACAGACACTGGTCG
	Mouse _ MMP2 _R	GCTGGTGCAGCTCTCATACT
Nos2	Mouse _ iNOS _F	TTCACAGCTCATCCGGTACG
	Mouse _ iNOS _R	GCCTAGGTCGATGCACAACT
Il6	Mouse _ IL-6_F	AGAGACTTCCATCCAGTTGCC
	Mouse _ IL-6_R	CCGGACTTGTGAAGTAGGGAA
β-actin	Mouse _ β-ACTIN _F	TAGGCGGACTGTTACTGAGC
	Mouse _ β-ACTIN _R	CTGCGCAAGTTAGGTTTTGTC
